# Cellular effects of glycine and trehalose air-polishing powders on human gingival fibroblasts in vitro

**DOI:** 10.1007/s00784-021-04130-0

**Published:** 2021-08-17

**Authors:** Jens Weusmann, James Deschner, Jean-Claude Imber, Anna Damanaki, Natalia D. P. Leguizamón, Andressa V. B. Nogueira

**Affiliations:** 1 Department of Periodontology and Operative Dentistry, University Medical Center of the Johannes Gutenberg University Mainz, Augustusplatz 2, 55131 Mainz, Germany; 2 Department of Periodontology, University of Bern, Freiburgstrasse 7, 3010 Bern, Switzerland; 3 Department of Diagnosis and Surgery, School of Dentistry At Araraquara, São Paulo State University, Araraquara, São Paulo, Brazil

**Keywords:** Air-polishing, Periodontology, Cell biology, Glycine, Trehalose

## Abstract

**Objectives:**

Air-polishing has been used in the treatment of periodontitis and gingivitis for years. The introduction of low-abrasive powders has enabled the use of air-polishing devices for subgingival therapy. Within the last decade, a wide range of different low-abrasive powders for subgingival use has been established. In this study, the effects of a glycine powder and a trehalose powder on human gingival fibroblasts (HGF) were investigated.

**Methods:**

HGF were derived from three systemically and periodontally healthy donors. After 24 h and 48 h of incubation time, mRNA levels, and after 48 h, protein levels of TNFα, IL-8, CCL2, and VEGF were determined. In addition, NF-κB p65 nuclear translocation and in vitro wound healing were assessed. Statistical analysis was performed by ANOVA and post hoc Dunnett’s and Tukey’s tests (*p* < 0.05).

**Results:**

Glycine powder significantly increased the expression of proinflammatory genes and showed exploitation of the NF-κB pathway, albeit trehalose powder hardly interfered with cell function and did not trigger the NF-κB pathway. In contrast to trehalose, glycine showed a significant inhibitory effect on the in vitro wound healing rate.

**Conclusion:**

Subgingivally applicable powders for air-polishing devices can regulate cell viability and proliferation as well as cytokine expression. Our in vitro study suggests that the above powders may influence HGF via direct cell effects. Trehalose appears to be relatively inert compared to glycine powder.

**Clinical relevance:**

With the limitations of an in vitro design, our study suggests that in terms of cell response, trehalose-based air-polishing powders show a reduced effect on inflammation.

## Introduction

The periodontium comprises the totality of the tissues that support the teeth, such as the alveolar bone, cementum, periodontal ligament, and gingiva. As the most common disease of these tooth-supporting structures, periodontitis is a chronic inflammatory biofilm-associated disease with socioeconomic, physiological, and psychological impact on patients [1]. Periodontitis is characterized by non-reversible damage of the periodontal tissues with a strongly variable inter-and intra-individual rate of progression. As a multifactorial disease, the dysbiosis of the subgingival microbiota plays a crucial role in periodontitis [2].

There is a clear consensus that the disintegration of the biofilm on the tooth root surface is an elementary component of any periodontal therapy and the core of the prevention measures [3–5]. That goal is primarily achieved by subgingival instrumentation with hand instruments and/or sonic or ultrasonic devices. After active periodontal therapy, including (if necessary) periodontal surgical approaches, the disease is monitored and controlled by regular professional plaque and calculus removal during “supportive periodontal therapy” (SPT). In this procedure, supra- and subgingival biofilm is removed mechanically, i.e., using hand instruments, sonic- or ultrasonic-operated scalers, and/or air-polishing devices, and the patient is trained in domestic oral care. The subgingival use of hand and sonic/ultrasonic instruments is associated with defects of the root surface. This risk increases over years of continuous SPT [6, 7]. Continuous supra- and subgingival removal of soft and hard deposits can result in massive loss of dental hard tissue. This may lead to hypersensitivity and damage of the pulp and even tooth loss in extreme cases.

Due to the protective effect on hard and soft tissues and relatively good patient acceptance, air-polishing devices with low-abrasive powders have increasingly gained access into the dental offices for the therapy of periodontitis and peri-implantitis. Air-polishing can be used adjuvant to mechanical subgingival instrumentation in primary therapy but also in SPT after completed primary treatment [8]. Various powders are commercially available for clinical use in periodontal and peri-implant patients. The powders must meet certain conditions such as non-cariogenity, and, if subgingivally applied, low abrasiveness and low inflammatory tissue response.

Since the 1980s, powders based on sodium bicarbonate with comparatively large grain size (250 µm) and coarse surface have been available. The use of air-polishing devices with sodium bicarbonate-based powders on exposed tooth roots or dentin can cause significant loss of tooth structure [9]. Due to these properties, sodium bicarbonate-based powders are unsuitable for subgingival applications.

The average particle size of glycine-based powders is significantly smaller than that of sodium bicarbonate (< 45 µm) [10]. Compared to sodium bicarbonate-based powders, powders based on glycine showed a reduction of root surface abrasiveness by approximately 80%. Glycine powder was shown to reduce the cultivable subgingival microbiota in periodontal pockets of 3–5 mm significantly better when compared to curettes [11–13]. Moreover, glycine has been shown to exert anti-inflammatory effects on keratinocytes [14].

Trehalose is a disaccharide that has shown anti-cariogenic properties in animal experiments as well as in in vitro [15]. Furthermore, it suppresses osteoclast differentiation in ligament-induced periodontitis in rats by inactivating RANKL induction [16]. The particle size is stated by the manufacturer to be 30 µm approximately. In a blinded randomized clinical trial, subgingival air-polishing with trehalose-based powder showed similar results to sonic instrumentation in SPT [17].

The introduction of low-abrasive powders has enabled the use of air-polishing devices for subgingival therapy. Within the last decade, more powders such as trehalose have been established. Mechanical efficiency of both glycine and trehalose powders has been shown in detail [10, 17].

Surprisingly, data regarding the effect of these powders on periodontal cells are scarce, although these powders get into direct contact with the respective cells. In addition, these cells are important for healing during periodontitis and peri-implantitis therapy. Therefore, in this study, the effects of the two different above-mentioned powders on human gingival fibroblasts were investigated. Based on earlier findings on glycine, the hypothesis was that glycine would also have anti-inflammatory and beneficial effects on fibroblasts.

## Materials and methods

### Cell culture and stimulation

Human gingival fibroblasts (HGF) were derived from healthy gingiva of patients who had undergone a wisdom tooth removal at the University Medical Center Mainz. The written informed consent of the donors was obtained. HGF were cultured in Dulbecco’s modified Eagle’s medium (DMEM, Invitrogen, Karlsruhe, Germany) supplemented with 10% fetal bovine serum (FBS, Invitrogen), 100 units of penicillin, and 100 μg/ml streptomycin (Invitrogen) at 37 °C in a humid atmosphere of 5% CO_2_. Cells were seeded between passages 3 to 6 on 6-well cell culture plates (50,000 cells/well) and grown until 80% confluence. One day before the start of the experiment, FBS concentration was reduced to 1%. HGF were stimulated with two different commercially available powders approved for subgingival application in air-polishing devices for 24 h and 48 h. A glycine-based powder (Perio-Mate Powder, NSK Europe, Eschborn, Germany) and a trehalose-based powder (Lunos Prophylaxepulver Perio Combi, Dürr Dental, Bietigheim-Bissingen, Germany) were used. Both powders were each dissolved in culture medium in a concentration of 1 g/20 ml, which corresponds to the powder/water ratio specified by manufacturers for emission from the air-polishing device. That concentration had already been used by other investigators [18].

### Real-time PCR

After 24 h and 48 h, cells were harvested and total RNA was extracted using a RNA extraction kit (RNeasy Protect Mini Kit, Qiagen, Hilden, Germany). The RNA concentration was determined by spectrophotometry (Nanodrop 2000, Thermo Fisher, Schwerte). The reverse transcription of 500 ng of RNA into cDNA was carried out using iScript™ Select cDNA Synthesis Kit (Bio-Rad, Munich, Germany) following the manufacturer’s protocol. Subsequently, the expressions of TNFα, IL-8, CCL2, VEGF, CASP3, PCNA, and GAPDH were analyzed by real-time PCR using the PCR thermal cycler (CFX96, Bio-Rad), SYBR Green PCR Master Mix (QuantiFast SYBR Green PCR Kit, Qiagen), and specific primers (QuantiTect Primer Assay, Qiagen). For this, 1 µl cDNA as template was mixed with 12.5 µl SYBR Green, 2.5 µl primers, and 9 µl nuclease-free water. The mixture was then heated at 95 °C for 5 min, followed by 40 cycles of denaturation at 95 °C for 10 s, and a combined annealing/extension step at 60 °C for 30 s. The data were analyzed by comparative threshold cycle method.

### ELISA

Protein levels of TNFα (RayBio Human TNF-alpha ELISA Kit, ELH-TNFα, RayBiotech, Norcross, GA, USA), IL-8 (RayBio Human IL-8 ELISA Kit, ELH-IL8, RayBiotech), CCL2 (Human CCL2/MCP-1 Quantikine ELISA Kit, DCP00, R&D Systems, Wiesbaden-Nordenstadt, Germany), and VEGF (RayBio Human VEGF-A ELISA Kit, ELH-VEGF, RayBiotech) in HGF cell culture supernatants were measured after 48 h of stimulation. Protocols were followed according to the manufacturer’s instructions for each specific kit. The concentration of TNFα, IL-8, CCL2, and VEGF was measured by spectrophotometry using a microplate reader (SynergyHT, BioTek Instruments, Winooski, VT, USA). Cell counting was performed using an automated cell counter (Luna™, Logos Biosystems, South Korea) to normalize the data by the cell number.

### Immunofluorescence

HGF were grown on plastic coverslips (Thermo Fisher Scientific) of 13-mm diameter and placed in 24-well plates in presence and absence of glycine or trehalose for up to 60 min. Next, cells were washed twice with PBS and fixed in 4% paraformaldehyde (Sigma-Aldrich, St. Louis, MI, USA) at pH 7.4 for 10 min. Subsequently, permeabilization of the cells was performed using 0.1% Triton X-100 (Sigma-Aldrich) for 5 min and then the cells were blocked with nonfat dry milk (Bio-Rad) for 1 h. After washing twice with PBS, cells were incubated with a rabbit anti-nuclear factor kappa B (NF)-κB p65 primary antibody (1:100, D14E12, Cell Signaling Technology, Danvers, MA, USA) at room temperature for 90 min. After the incubation step, cells were washed again twice with PBS and incubated with a CY3-conjugated goat anti-rabbit IgG secondary antibody (1:1000, ab6939, Abcam, Berlin, Germany) at room temperature for 45 min. The NF-κB p65 nuclear translocation was observed by using the ZOE™ Fluorescent Cell Imager (Bio-Rad). Untreated cells served as control.

### In vitro wound healing

To analyze the effect of glycine or trehalose on in vitro wound healing, a well-established in vitro wound healing model was used as in previous studies (Memmert et al., 2018, Nokhbehsaim et al., 2011). HGF were cultivated on 35 mm plastic culture dishes and grown up to 100% confluence. One day after reduction of the FBS concentration, a 3–4 mm wide wound was inflicted using a sterile 100 µl pipette tip, resulting in cell-free areas in the cell monolayers. Subsequently, all non-adherent cells were removed by several washing steps with PBS and later with DMEM. The injured monolayers were then stimulated with glycine or trehalose for 48 h. Using a JuLI™ Br and the PC software JuLI™ Br (both NanoEnTek, Seoul, Korea), wound closure was documented and evaluated over time. For data analysis, wound healing rates were calculated.

### Statistics

The statistical analysis was performed using the software GraphPad Prism version 9.0 (GraphPad Software Inc., San Diego, CA, USA). Data were presented as mean values and standard errors of the mean (SEM). Normal distribution was tested. Significant differences between groups were determined using *t*-test or ANOVA followed by the post hoc Tukey’s or Dunn’s tests. The significance level was considered at *p* < 0.05.

## Results

### Effect of glycine and trehalose on proinflammatory markers in HGF

**Fig. 1:**
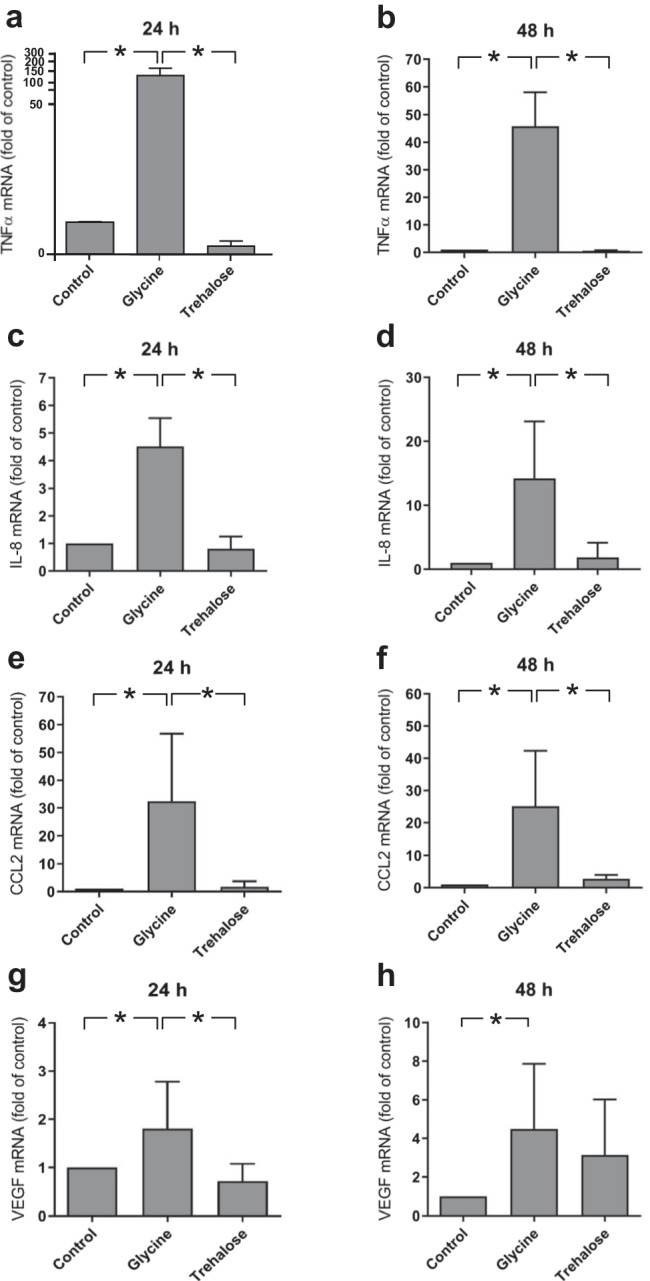
Expressions of TNFα (**a**–**b**), IL-8 (**c**–**d**), CCL2 (**e**–**f**), and VEGF (**g**–**h**) in HGF in presence of glycine and trehalose at 24 h and 48 h. Unstimulated cells served as control. Mean ± SEM (*n* = 9). *Significant (*p* < 0.05) difference between groups

Glycine enhanced the expression of the evaluated proinflammatory markers, as analyzed by real-time PCR (Fig. 1). TNFα, IL-8, and CCL2 expressions were significantly (*p* < 0.05) upregulated in glycine-treated HGF compared to control and trehalose-treated cells at 24 h and 48 h (Fig. 1a–f). The expression of VEGF was significantly (*p* < 0.05) increased in the glycine group compared to the control group at 24 h and 48 h and the trehalose group at 24 h (Fig. 1g–h).

**Fig. 2:**
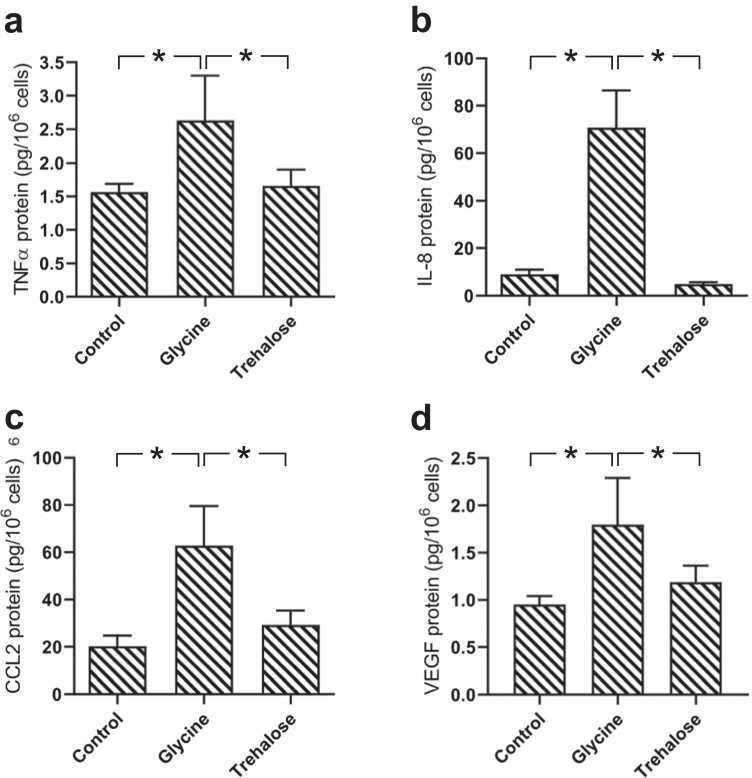
Protein levels of TNFα (**a**), IL-8 (**b**), CCL2 (**c**), and VEGF (**d**) in HGF in presence of glycine and trehalose at 48 h, as analyzed by ELISA. Unstimulated cells served as control. Mean ± SEM (*n* = 18). *Significant (*p* < 0.05) difference among groups

The stimulatory effect of glycine on HGF was also demonstrated at the protein level, as analyzed by ELISA (Fig. 2). Increased protein levels of all proinflammatory markers were observed for glycine-treated cells. Significant (*p* < 0.05) higher protein levels of TNFα, IL-8, CCL2, and VEGF were found in the glycine group compared to the control and trehalose groups (Fig. 2a–d).

### Exploitation of the NF-κB pathway by glycine

**Fig. 3:**
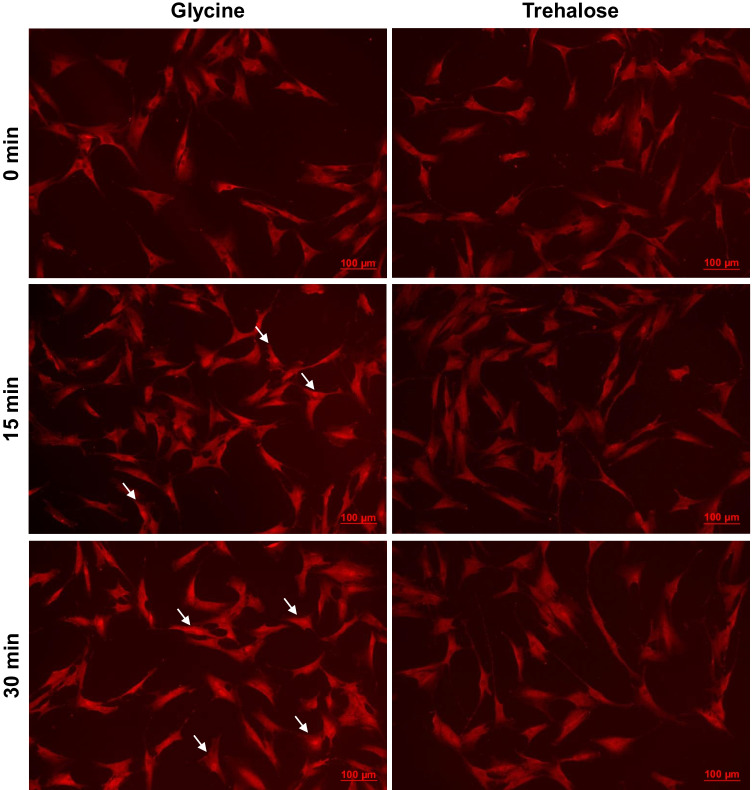
NF-κB (p65) nuclear translocation in HGF exposed to glycine or trehalose over time. Representative images from one out of three experiments are shown. Indicator arrows show some cells presenting nuclear translocation of p65 at 15 min and 30 min

Since glycine induced an increase in the expression of proinflammatory mediators which are known to be triggered by NF-κB, we next evaluated p65 nuclear translocation in HGF stimulated with glycine. As evidenced by immunofluorescence microscopy, glycine-stimulated HGF showed nuclear translocation of p65 at 15 min and 30 min (Fig. 3). In contrast, trehalose showed no or little exploitation of NF-κB signaling (Fig. 3).

### Regulation of apoptosis and proliferation markers by glycine and trehalose in HGF

**Fig. 4:**
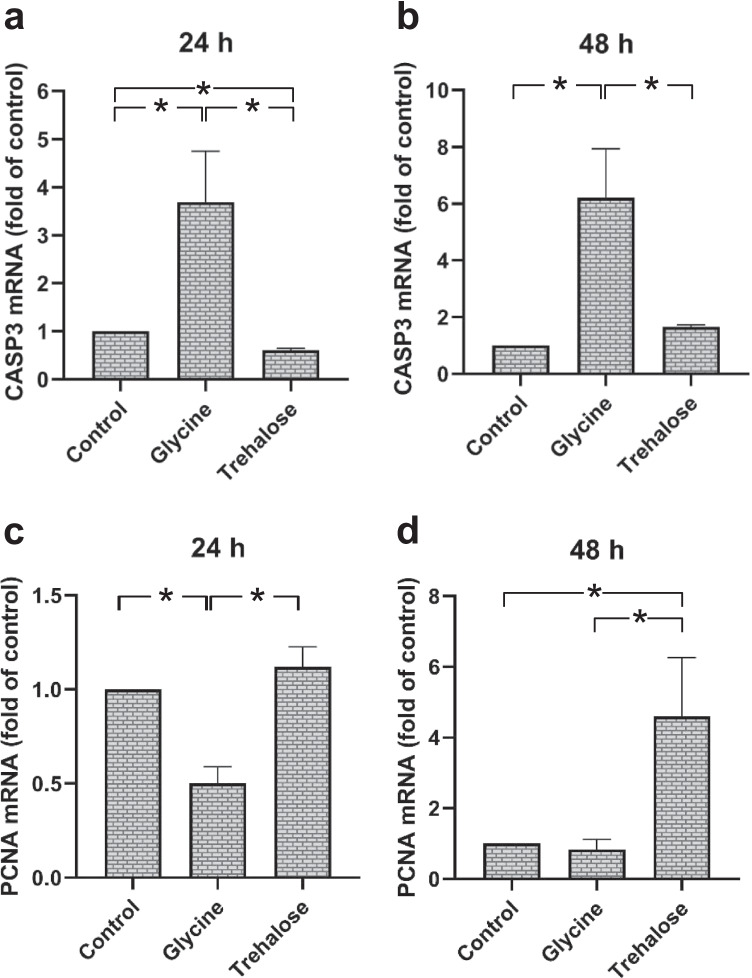
Expressions of CASP3 (**a**) and PCNA (**b**) in HGF in presence of glycine or trehalose at 24 h and 48 h. Unstimulated cells served as control. Mean ± SEM (*n* = 9). *Significant (*p* < 0.05) difference among groups

Glycine induced a significant (*p* < 0.05) increase in the expression of caspase-3, a marker of apoptosis, compared to control and trehalose groups at 24 h (Fig. 4a). At 48 h this increase was even higher compared to 24 h (Fig. 4a–b). Next, we investigated the cell proliferation marker PCNA. Decreased expression of PCNA was observed in the glycine group compared to control and trehalose groups at 24 h (Fig. 4b). The trehalose-stimulated HGF showed an upregulation of PCNA compared to control and glycine groups at 48 h (Fig. 4d).

### Effect of glycine and trehalose on wound healing

**Fig. 5:**
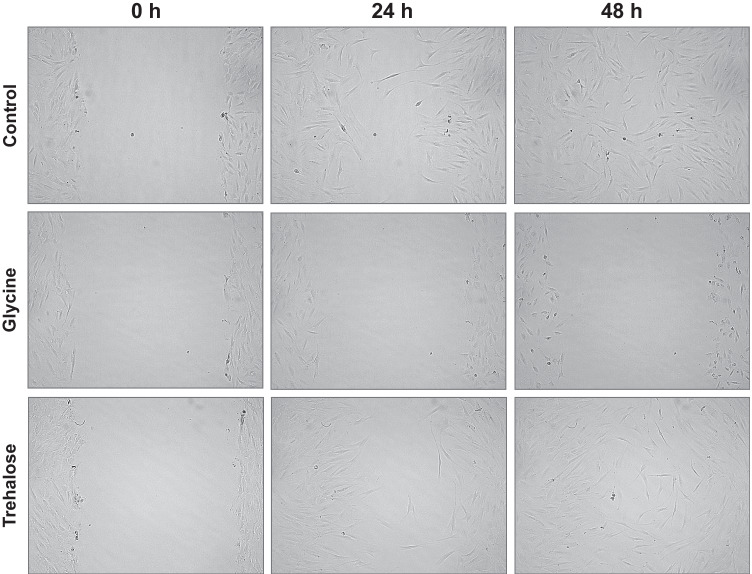
Wound closure of HGF cell monolayers in presence or absence of glycine and trehalose at 0 h, 24 h, and 48 h. Images from one representative donor are shown

**Fig. 6:**
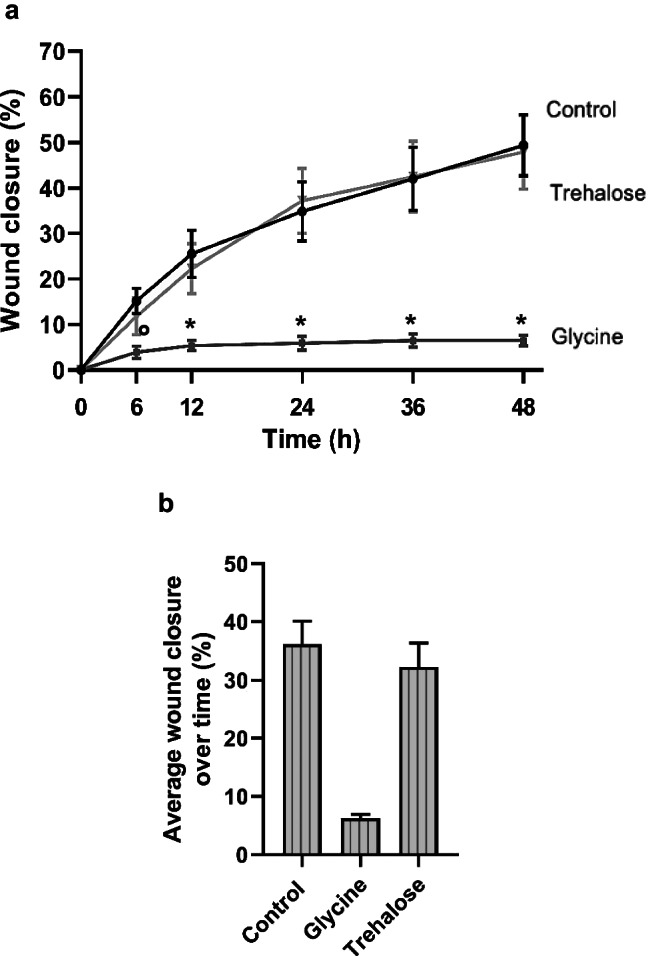
**a** Wound closure of HGF in the presence of glycine and trehalose over 48 h. Wound closure, i.e., the percentage of cell coverage of the initially cell-free zones created by wounding, was analyzed by live-cell imaging. Mean ± SEM. *Significant (*p* < 0.05) difference from all groups; ∘ significant difference to control. **b** Average wound closure of HGF in presence of glycine or trehalose is shown. Mean ± SEM; *Significant (*p* < 0.05) difference among groups.

No significant difference between control and trehalose was detected. After 24 h, the wound closure rate demonstrated a similar pattern in both groups (Figs. 5 and 6a). In contrast, the wound filling rate of glycine-treated HGF was significantly (*p* < 0.05) lower compared to the control at all time points and trehalose at 12 h, 24 h, 36 h, and 48 h (Figs. 5 and 6a). The average wound closure summarizing the data obtained from all time points for each group revealed a wound filling percentage of 36.1% for the control group, 32.3% for the trehalose group, and 6.2% for the glycine group (Fig. 6b).

## Discussion

In the present in vitro study, two different powders for air-polishing were investigated. Our results show for the first time that the glycine-containing powder has proinflammatory and proapoptotic effects, whereas the trehalose-based powder is largely inert. Moreover, unlike trehalose, glycine exerted antiproliferative and inhibitory effects on wound healing. Our data suggest that in terms of cell response, trehalose is more beneficial. The question of the clinical relevance of these findings remains unanswered; our in vitro results can only be considered as a guide in this regard. To our knowledge, there is only one study that has investigated the cellular response on air-polishing powders so far. In this study, the effects of two different glycine-based powders on epithelial cells, periodontal ligament fibroblasts, and gingival fibroblasts were evaluated [18]. One of the glycine-based powders interfered with the proliferation of HGF while the other did not. The authors suggested that these findings may be due to the different powder compositions [18]. Therefore, their results are partially in agreement with our data. It must be mentioned that silicates and possibly other free-flowing aids are added in various compositions to these powders in traces that might have influenced the results. In our study, a significant increase in the expression of caspase-3, a marker of apoptosis, and a significant decrease in the expression of the proliferation marker PCNA was found in response to the glycine-based powder. It must be considered that the time of incubation with the powders was shorter in the study by Sygkounas et al. [18]. It should be noted that the exposure time in the gingival sulcus may indeed be less than 48 h. Therefore, the transferability of the results of our in vitro study to clinical events is not completely possible in this respect. We have found that glycine caused a significant increase in the expression and synthesis of a great number of well-known inflammatory mediators, such as TNFα [19]. This cytokine is involved in a diversity of disease processes such as tissue destruction [20]. It initiates the promotion of rolling and adhesion of neutrophils to blood vessel walls by upregulation of adhesion molecules, resulting in extravasation [21]. Moreover, TNFα stimulates the production of different chemokines and therefore plays an important role in cell migration to infected and inflamed sites [21, 22]. In periodontitis, TNF is a main contributor to periodontal damage in periodontal tissue destruction [23].

We also found an increased expression and synthesis of IL-8 and CCL2. Chemokines, such as IL-8 and CCL2, play an important role during inflammatory processes and are produced by immunoinflammatory and structural cells including gingival fibroblasts [21]. CCL2 is involved in the recruitment of immunoinflammatory cells such as monocytes, macrophages, and lymphocytes [24]. It has been shown in previous studies that CCL2 levels are upregulated in gingival crevicular fluid from periodontally diseased sites compared to healthy sites [25]. Moreover, microbial stimuli and mechanical signals are capable of regulating CCL2 in periodontal cells and tissues [25, 26]. In our study, the glycine-based powder was the only powder that significantly elevated the gene expression of CCL2 after 24 and 48 h. For IL-8, the expression pattern resembles that of TNFα after 24 h with a statistically significant elevation in the glycine-based powder group compared to the other groups. The enhanced expression of proinflammatory genes such as TNFα, IL-8, and CCL2 in our study concurs very well with our data on the cell viability mentioned above. Regarding IL-8, we found a 4.5-fold and 15-fold increased expression after 24 h and a 48 h, respectively. CCL2 was even increased by 20-fold and 30-fold at 24 h and 48 h, respectively. These findings underline the strong proinflammatory action of the glycine-based powder investigated in our study.

VEGF, as a promoter of angiogenesis, was also significantly increased by glycine as compared to the control group. This could suggest a possible role of the glycine powder in tissue repair and regeneration. However, in our study, glycine-based powder interfered negatively with in vitro wound healing. In addition, like TNFα, VEGF has been shown to be increased in periodontitis, indicating a possibly proinflammatory and destructive role in periodontal diseases [27].

A study by Schaumann et al. on keratinocytes has also shown an immunoregulatory role for glycine. In this study, glycine led to an increased expression of IL-6, which concurs with our findings on proinflammatory mediators [14].

Anti-inflammatory, immunomodulatory, and cytoprotective effects of glycine have each been linked to different intracellular signaling pathways [28]. We found that glycine-based powder activates the NF-kB pathway. Whether other pathways are also involved in the proinflammatory actions of glycine has to be elucidated in further studies. Whether inflammation is possibly only harmful or, on the contrary, plays a positive role in wound healing or stimulation of phagocytosis remains to be clarified.

In our study, we were able to show that glycine-based powder inhibited the in vitro wound healing. The in vitro wound healing rate depends not only on apoptosis and migration but also on cell proliferation. In this respect, it may be that the inhibitory effect of glycine on the in vitro wound healing was mediated, at least in part, by inhibition of proliferation.

To the knowledge of the authors, there is no literature about the in vitro effects of trehalose on HGF. In addition, scientific reports on the clinical efficacy of trehalose are sparse. Interestingly, anti-resorptive effects of trehalose have been detected in bone cells [16]. Regarding the possible role of trehalose in dentistry, most of the pertinent literature deals with the anti-cariogenic effects [15].

As mentioned above, the cellular response to trehalose was very different as compared to glycine in our study. With regard to the proinflammatory and angiogenetic cytokines, no significant difference between trehalose and control was found. While the clinical efficacy of the trehalose-based powder has been demonstrated, data on the cellular response to trehalose are still limited [17]. It has been shown that proinflammatory cytokines could be inhibited by trehalose in mouse peritoneal macrophages [29]. In a rat periodontitis model, trehalose was able to suppress osteoclast differentiation by inactivation of RANKL [16]. These findings are in line with our observations that trehalose-based air-polishing powders do not have a proinflammatory effect. These results are in agreement with our observation, that the NF-κB pathway, which is associated with inflammation, is not triggered by trehalose. Trehalose might have an inhibiting effect on inflammatory cytokine production in mouse macrophages via the trehalose receptor T1R3 by inhibiting the dismantling of IkappaB-alpha [29]. Moreover, trehalose is known to be a significant modulator of autophagy and can therefore influence inflammation positively [30]. It remains unclear to what extent trehalose could also exert a negative effect on the oral cavity. It has been shown that dietary trehalose can contribute to the virulence of *Clostridium difficile* diseases [31]. This microbiological aspect should be explored in the future. Further studies should also assess the actions of trehalose on autophagy in periodontal cells.

## Conclusion

In summary, our in vitro study showed for the first time that the glycine-containing powder had proinflammatory and proapoptotic effects, whereas the trehalose-based powder was largely inert. In addition, unlike trehalose, glycine exerted antiproliferative and inhibitory effects on wound healing. Therefore, our study suggests that in terms of cell response, trehalose-based air-polishing powder might be more beneficial. Due to the in vitro nature of this study, these results are not necessarily clinically applicable. Whether findings from this study have an impact on clinical results after periodontal therapy comparing glycine- and trehalose-based powders has to be investigated.
